# Behind the Adaptive and Resistance Mechanisms of Cancer Stem Cells to TRAIL

**DOI:** 10.3390/pharmaceutics13071062

**Published:** 2021-07-10

**Authors:** Adriana G. Quiroz-Reyes, Paulina Delgado-Gonzalez, Jose Francisco Islas, Juan Luis Delgado Gallegos, Javier Humberto Martínez Garza, Elsa N. Garza-Treviño

**Affiliations:** 1 Department of Biochemistry and Molecular Medicine, Faculty of Medicine, Autonomous University of Nuevo Leon, San Nicolás de los Garza 64460, Mexico; adri.quiroz.ry@gmail.com (A.G.Q.-R.); paulina.delgadogn@uanl.edu.mx (P.D.-G.); jose.islasc@uanl.mx (J.F.I.); jdelgado.me0174@uanl.edu.mx (J.L.D.G.); 2 Department of Human Anatomy, Faculty of Medicine, Autonomous University of Nuevo Leon, San Nicolás de los Garza 64460, Mexico; javier.martinezgr@uanl.edu.mx

**Keywords:** TRAIL, cancer stem cells, TRAIL resistance, angiogenesis

## Abstract

Tumor necrosis factor (TNF)-related apoptosis-inducing ligand (TRAIL), also known as Apo-2 ligand (Apo2L), is a member of the TNF cytokine superfamily. TRAIL has been widely studied as a novel strategy for tumor elimination, as cancer cells overexpress TRAIL death receptors, inducing apoptosis and inhibiting blood vessel formation. However, cancer stem cells (CSCs), which are the main culprits responsible for therapy resistance and cancer remission, can easily develop evasion mechanisms for TRAIL apoptosis. By further modifying their properties, they take advantage of this molecule to improve survival and angiogenesis. The molecular mechanisms that CSCs use for TRAIL resistance and angiogenesis development are not well elucidated. Recent research has shown that proteins and transcription factors from the cell cycle, survival, and invasion pathways are involved. This review summarizes the main mechanism of cell adaption by TRAIL to promote response angiogenic or pro-angiogenic intermediates that facilitate TRAIL resistance regulation and cancer progression by CSCs and novel strategies to induce apoptosis.

## 1. Introduction

Apoptosis, or programmed cell death, is a naturally occurring mechanism that eliminates damaged cells. Intracellular signals from mitochondria or by ligands that bind to receptors on the cell membrane, such as Fas, tumor necrosis factor (TNF)-α/TNF receptor 1, Apo-3 ligand/death receptor (DR) 3, and TNF-related apoptosis-inducing ligand (TRAIL), can initiate apoptosis [1,2]. Intrinsic and extrinsic pathways can activate apoptosis. The extrinsic pathway is induced by signals that activate cell surface death receptors, such as the binding of TNF-α to TNF-R1. Meanwhile, an example of an intrinsic pathway can be seen as induced by the B cell lymphoma 2 (Bcl-2)-regulated mitochondrial pathway, leading to a release of cytochrome c, which associates with apoptotic protease activating factor 1 (APAF-1) and pro-caspase-9, forming the apoptosome, leading to the activation of caspase-9 [3]. Thus, apoptosis plays an important role in physiological processes, including the development of cardiovascular and neurological diseases and malignancies [4]. 

TRAIL was discovered in the last decade of the 20th century [5]. It is a TNF-related type 2 transmembrane protein encoded by the NFSF10 gene located on human chromosome 3 at 3q26. In the organism, TRAIL is part of the mechanism by which the immune system reacts against malignant cells, inducing apoptosis with minimal cytotoxicity toward normal cells [6]. The TRAIL protein consists of 281 amino acids of 33 KDa and, in its fully glycosylated form, 41 KDa. However, TRAIL is cleaved at the 114 amino acid position by cysteine proteases to produce soluble TRAIL, a protein of 24 KDa called sTRAIL. For TRAIL to fully activate, an insertion of 12–16 amino acids in the receptor-binding site is required [7]. Physiologically, TRAIL is secreted by different tissue cells in the spleen, lung, prostate, placenta, kidney, cytotoxic T cells, and natural killer cells. Monocytes and dendritic cells can express TRAIL on their surface after stimulation with interferon-β (IFN-β), which boosts antitumoral activity. In addition, there is a trace of sTRAIL in blood plasma (approximately 100 pg/mL) [8]. 

TRAIL can bind to five different receptors. DR4 and DR5 are death receptors (TRAIL-R1 and TRAIL-R2). Decoy receptors (DcR1 or DcR2, also TRAIL-R3 and TRAIL-R4) are anti-apoptosis receptors. The first receptors are overexpressed in tumors, and the latter are expressed mainly in normal cells [9]. The difference between both receptor types is the lack of cytoplasmic domains required for apoptosis activation. Another receptor discovered to bind TRAIL is osteoprotegerin (OPG). OPG is a soluble receptor that inhibits TRAIL apoptosis [10]. DR5 has been reported to be more efficient in apoptosis induction. However, DR5 nuclear localization in tumor cells is a signal of resistance to TRAIL [11]. 

DR4 and DR5 are transmembrane proteins with several cysteine-rich domains (CRDs) in their extracellular region, a single transmembrane domain, and a death domain (DD) in their intracellular region. DDs are homotypic protein modules organized in six alpha-helices that act as binding sites for other proteins and communicate the apoptotic signal to the cell interior [7]. After TRAIL binding to DR4 and DR5 occurs, their trimerization initiates by the interaction of the DD and the Fas-associated death domain (FADD), inducing TRAIL receptors that expose the death effector domain (DED) and the formation of the death-inducing signaling complex (DISC) [3]. Next, the DISC recruits pro-caspase-8 and cleaves it. Cleaved caspase-8 decreases the membrane potential and converts Bid to truncated Bid (tBid). Then, tBid, p53, Noxa, Puma, and Bax form the pore-forming complex in the mitochondria outer membrane [12]. After pore formation, cytochrome C is released into the cytoplasm, interacting with dATP. Pro-caspase-9 is then recruited to Apaf-1 to form the Apaf-1/caspase-9 apoptosome [13]. Active caspases are proteolytic proteins that bench to cytosolic and nuclear targets; caspases cause cleavage of actin filaments of the cytoskeleton, the inhibitor of caspase-activated DNase (ICAD) that avoids activation of caspase-activated DNase (CAD) that destroys DNA [12]. 

This mechanism of cell death, which has been widely studied in the context of cancer and other diseases, is efficient. Many basic and clinical studies have demonstrated a relevant role in selectively inducing apoptosis, and in tumor cells, this has been demonstrated. Therefore, apoptosis continues to be a therapeutic target that needs to be studied.

## 2. Recombinant TRAIL

Recombinant versions of human APO2L/TRAIL have been developed and used in clinical trials due to their potential as antitumoral therapy. However, there has been some limitation in their use as an anticancer drug because of their short half-life in blood, fast elimination, and resistance by cancer cells [10]. Dulanermin is a recombinant non-tagged TRAIL used in clinical trials. However, this protein has not shown important therapeutical benefits due to poor efficiency binding to TRAIL receptors [14]; another form of Dulanermin, Apo2L.XL, presents higher pro-apoptotic activity by artificial cross-linking [7].

Tumoral cells can generate TRAIL resistance by downregulation of DR4 and DR5 and inhibition of the CD95/Fas domain [6]. Moreover, the signal received in TRAIL receptors can be switched to activate non-canonical signaling, inducing pro-inflammatory, pro-survival, and proliferation characteristics. This signal involves a complex integrated by receptor-interacting serine/threonine protein kinase 1 (RIPK19), tumor necrosis factor (TNF) receptor-associated factor 2 (TRAF2), and TNF receptor-associated death domain (TRADD), activating pro-tumor pathways, such as IkB/NF-kB, MAPK/ERK, STAT3, PI3K, Akt, JAK2, and Src [10]. 

The development of different recombinant TRAIL therapies has gained importance as a new strategy for reducing cancer progress. As mentioned before, TRAIL receptors have been discovered mainly in cancer cells; however, their overexpression has been reported in a particular population of cancer cells in tumors called cancer stem cells (CSCs).

## 3. Cancer Stem Cells and TRAIL

CSCs are a subpopulation of cells that represent a low percentage within the tumor niche. These cells have pluripotency, self-renewal, and tumorigenic properties, such as invasiveness, plasticity, and maintenance, and are the main cause of chemoresistance and cancer relapse. Several CSC markers have been identified, such as homing cell adhesion molecule (CD44) [15], aldehyde dehydrogenase (ALDH) [15], CD326 [16], and CD133 [17]. Although some of these markers are useful for identification and therapeutic targets, they are further found in normal stem cells and are not specific [16].

Epithelial–mesenchymal transition (EMT) is a process associated with the stemness of cancer cells, which is critical during cancer progression. This process of EMT implicates the conversion of epithelial cells into a mesenchymal phenotype with loss of cell–cell junctions, altering cell–ECM interactions and cytoskeletal organization [18]. EMT mediators can stimulate the increased malignancy associated with the CSC phenotype, such as migration and invasion by protein expression and activation of transcription factors. These factors include SNAI1 and SNAI2 (Snail and Slug), ZEB1 (dEF1/TCF8), and ZEB2 and Twist. Others are Prrx1, Sox4, Sox9, Klf4, and members of the AP-1 (Jun/Fos) family [19]. Pathways involved in EMT include transforming growth factor-beta (TGF-β), bone morphogenetic protein (BPM), receptor tyrosine kinase (RTK), Wnt/β-catenin, Notch, Hedgehog, signal transducer and activator of transcription 3 (STAT3), extracellular matrix (ECM), and hypoxia [20]. 

Genes of stem lineage Oct-4 and Nanog upregulate the process of EMT by binding to the promotors of Zeb1, Zeb2, Twist1, Prrx1, and miR-21. In addition, Sox2 increases slug expression, activating the STAT3/ hypoxia-inducible 1alpha (HIF-1α) signaling pathway, inducing EMT and promoting metastasis [20,21,22]. Altogether, these factors improve the protection of senescence and apoptosis and regulate cell progression and resistance to chemotherapy and radiotherapy, reducing E-cadherin expression, which drives to a mesenchymal state. In the tumor microenvironment, cancer cells secrete factors, such as TGF-β, hepatocyte growth factor (HGF), and platelet-derived growth factor (PDGF), activating changes in EMT [23]. Moreover, TGF-β is a major inducer of EMT since it can interact with other growth factors, such as epidermal growth factor (EGF), to influence the malignant transformation of CSCs and tumor-associated stromal fibrosis [23]. Once activated, EMT increases the expression of genes involved in stemness and stem cell markers. Additionally, in breast cancer, EMT increases stem cell phenotypes, such as CD44+/CD24- markers. EMT markers, such as E-cadherin, β-catenin, Snail, and Vimentin, correlate with CD133 expression, invasion, and metastasis of CSCs [20].

## 4. TRAIL Resistance Mechanism

EMT promotes TRAIL resistance and silencing of E-cadherin, which inhibits apoptosis due to the lack of efficient DISC assembly by ligated TRAIL receptors [10]. CD133 is a cell marker commonly expressed in CSCs, such as in colorectal cancer (CRC) and glioma [13]. Other CSC markers include CD44, nestin, and sox-2, usually co-expressing with CD133 [9,23]. CD133-positive cells present high ATP-binding cassette transporter (ABCG5) expression related to chemoresistance [12]. For this reason, these cells are novel targets for cancer therapy [12]. Embryonic pathways, such as Notch, Wnt, Hedgehog, and Hippo, are overactivated in CSCs to maintain their stem cell characteristics [24]. The Wnt signaling pathway participates in the chemoresistance of CD133-positive cells in CRC; thus, it is also considered a potential target [25]. Moreover, CD133 activates the PI3K pathway, and this, Akt, whose activation leads to upregulation of anti-apoptosis factors, such as BCL-2, BCL-XL, and MCL-1, decreases the pro-apoptotic factors Bid, Bax, and Bim [12]. 

**Figure 1:**
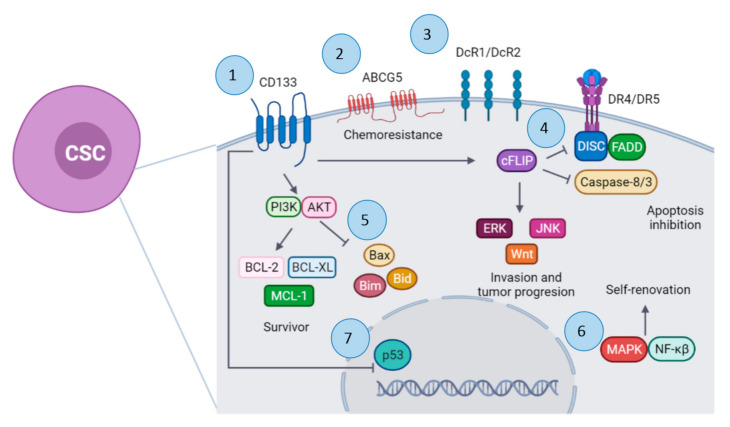
Mechanism of apoptosis resistance by cancer stem cells (CSCs). 1. CD133 activates the PI3K/AKT pathway. 2. Overex-pression of ATP-binding cassette transporter (ABCG5). 3. Overexpression of decoy receptors (DcR1/DcR2) and down-regulation of DR4/DR5 receptors. 4. Overexpression of cFLIP protein. 5. Downregulation of pro-apoptotic factors. 6. Upregulation of MAPK and NF-kβ pathways. 7. Downregulation of p53. Created with BioRender.com (accessed on 25 May 2021).

Although TRAIL is a promising anticancer therapy, which can induce apoptosis in tumoral cells instead of normal cells, some CSC tumoral cells develop resistance to TRAIL (Figure 1) [26]. However, TRAIL treatment resistance has been developed by a variety of cancers [27]. Another TRAIL resistance mechanism associated with downregulation of the dead receptors DR4 and DR5 is overexpression of the decoy receptors DcR1 and DcR2 and overexpression of apoptosis inhibitors, such as cFLIP. It is suggested that activation of NF-kB could upregulate DR5 expression [9]. In turn, activation of the NF-kB pathway by TRAIL is associated with improvement of tumor growth, clonal expansion, and CSC signaling [6]. TRAIL-R2 or DR5 promotes invasion and metastasis of KRAS-mutated cancers by activating Rac1/phosphatidylinositol-3-kinase (PI3K) signaling. This metastasis process is cell-autonomous and mediated by the membrane-proximal domain (MDP) of the receptor [28]. In addition, it is reported that TRAIL-R downregulation and apoptosis resistance are mediated by signals, such as Src, Talin, PI3K, and MAP. Phosphatase and tensin homolog (PTEN) protein negatively regulates the PI3K/AKT/mTOR pathway, working as a tumor suppressor gene, and EMT [29].

Moreover, the molecule ONC201/TIC10 can induce apoptosis by the TRAIL pathway, showing great results in vitro and in vivo. It is currently being used in phase I/II clinical trials for advanced-stage cancer, such as breast cancer, colon cancer, and glioblastoma. The mechanism of ONC201/TIC10 is the inactivation in CSCs of Akt and ERK signaling, inducing Foxo3 nuclear translocation and transcription of TRAIL, independent of the p53 status. In addition, this leads to the expression of the TRAIL receptor DR5. This molecule improves the half-life, distribution, route of administration, and activity of recombinant TRAIL and TRAIL-agonist antibodies, the main problems in its clinical application [30].

CD133-positive cells can also develop TRAIL resistance by overexpression of FLICE-like inhibitory protein (FLIP), inhibiting the DISC ensemble and TRAIL apoptosis. Moreover, p53 protein expression has an inverse relationship with CD133 expression [12]. cFLIP overexpression is also associated with TRAIL resistance in cancer cells. cFLIP inhibits caspase-8 and caspase-3 activation [9]. The expression of FLIP, a caspase-8 inhibitor, is higher in CD133-positive cells than in CD133-negative cells. CD133 upregulates FLIP expression, and this protein inhibits autophagy and activates ERK, JNK, ERK, and Wnt pathways. FLIP also inhibits FADD [12,26]. According to some studies, suppression of cytoplasmic cFLIP and elevated nuclear cFLIP levels are associated with regulating the Wnt pathway, which impacts the maintenance of CSCs. Inhibition of cFLIP further reduces beta-catenin levels and inhibits Wnt target gene expression, whereas overexpression of nuclear cFLIP promotes Wnt target gene expression [31]. In addition, downregulating Wnt/β-catenin signaling impacts TRAIL sensitivity and reduces EMT [32,33]. TRAIL resistance is also associated with activation of self-renewing pathways by mitogen-activated protein kinases (MAPKs) and NF-kβ, both negatively regulated by PTEN. The high expression of PTEN correlates with a better TRAIL response in tumors and with the reverse process of EMT (mesenchymal–epithelial transition (MET)) [34].

Although there are CSCs resistant to TRAIL, some research groups have reported that CSCs are susceptible to TRAIL activity after stimulation with small-molecule compounds, such as CDDP, etoposide, PS-341 (bortezomib), tunicamycin, rottlerin, brandisianins, sodium butyrate, and inostamycin [35]. Other natural compounds, such as kurarinone, icaritin, and withanolide E, are reported to downregulate cFLIP expression and TRAIL-resistant cancer cell sensitization to TRAIL-induced apoptosis. Natural compounds, such as silibinin, gingerol, and indomethacin, are reported to possess both mechanisms of sensitizing TRAIL-resistant cancer cells. The most relevant results are summarized in Table 1. A TRAIL-sensitive phenotype can be observed in different types of cancers and under different conditions. TRAIL can induce contrasting effects in tumoral cells, mainly controlled by the TME. The combination of TRAIL with some compounds prevents CSC TRAIL resistance and induces its elimination as sulforaphane [6]. Additional treatment with cisplatin combined with recombinant TRAIL could restore the expression of death receptors and Fas domain activity [6]. Dickkopf-1 (DKK-1) diminishes the expression of CD133, and consequently, proliferation, migration, and invasion of cancer cells diminish [25]. Agonists of TRAIL receptors have not presented enough efficacy due to the complexity of TRAIL signaling [10].

## 5. Microenvironment and TRAIL Activity

The interaction of cells in the TME and CSCs can change the signaling mechanism of TRAIL in a tumor, leading to cell death or disease progression [46]. An antitumor TME is made by normal fibroblasts, dendritic cells (DC), natural killer (NK) cells, cytotoxic T cells, and M1 tumor-associated macrophages (TAMs) with the release of pro-inflammatory cytokines [10]. In contrast, a protumor TME includes M2 TAMs producing anti-inflammatory cytokines; myeloid-derived suppressor cells (MDSCs); regulatory T cells (Tregs) and B cells; cancer-associated fibroblasts (CAFs); and TIE2-expressing monocytes, mast cells, pericytes, and endothelial cells. In addition, neutrophils and T helper cells present both roles, pro- and anti-tumorigenic activity [47]. Different factors can regulate the production and secretion of TRAIL by several immune cells from the innate and adaptative immune systems. On the other hand, these similar factors can regulate the expression of membrane bound TRAIL and its receptors in the cellular microenvironment from components in TME, such as cells, cytokines, pH, oxygen levels, and matrix components [7]. For example, cytokines as IFNs can activate TRAIL transcription by the IRF1/STAT complex. TRAIL and TRAIL-R transcription is also mediated by stress-induced factors, such as nuclear factor of activated T cells (NFAT), Forkhead Box (FOX) proteins, NF-kB, C/EBP homologous protein, activator protein 1 (AP1), and p53 [10]. 

Physiologically, the TRAIL/TRAIL-R system regulates the homeostasis of adaptative immune cells by inducing apoptosis of aberrantly activated T cells. NK cells eliminate aberrant tumor cells by granule release (perforin/granzyme) in the innate immune system. This release is dependent on membrane receptor interactions involving FasL, TNFα, and TRAIL. TNFα increases TRAIL expression in mesenchymal stem cells (MSCs), inhibiting tumor growth by apoptosis induction of cancer cells [7]. In addition, DNA released from MSCs could act as damage-associated molecular patterns (DAMPs) that via TLR3-dependent NF-kB feed-forward loop further increase TRAIL expression on MSCs. Furthermore, TNFα-activated MSCs also produce IFNβ due to DNA/RNA released from apoptotic cells, thus enhancing TRAIL expression [10]. The activation of NK cells by IL12 generates IFNγ, which enhances TRAIL expression [48].

Monocytes can also express TRAIL and target TRAIL-R in tumoral cells. It is seen that IFNα increases the release of soluble TRAIL by monocytes and promotes apoptosis of tumoral cells [27,49]. Moreover, macrophages secrete matrix metallopeptidase 12 (MMP12), which can mimic TRAIL and induce apoptosis in tumor cells [50]. TRAIL also induces CD14 and CD11b expression in monocytes, promoting its M1 differentiation and its phagocytic capacity and antitumor activity [51]. In addition, by TRAIL stimulation, macrophages produce pro-inflammatory cytokines IL1β, IL6, and TNFα in an NF-kB-dependent way [10]. 

As a component of the ECM, elastin microfibril interface-located protein 2 (EMILIN2) can bind to TRAIL receptors DR4 and DR5, inducing clustering and co-localization on lipid rafts from cell membrane, and then induce activation of apoptosis [10]. Hypoxia factors, such as HIF-1α, are associated with PKCε down-modulation, which acts as a key molecular event that promotes apoptosis by TRAIL in hypoxic tumor cells. In addition, the expression of vascular cell adhesion molecule-1 (VCAM-1) by tumors has been proposed as an immune escape mechanism and improves metastasis. VCAM-1 and a4 integrin interaction promotes T cell migration away from the tumor, reducing CD8+ T cell infiltration [52]. Cytotoxic T cells (CTLs) are the main effectors of the adaptative immune system against tumor cells, expressing TRAIL and TRAIL-R. This TRAIL expression is stimulated by interaction with TRAIL receptors on tumoral cells [10]. In addition, IFNα stimulates CTLs to increase TRAIL expression. Dendritic cells (DCs) participate in innate and adaptative immunity, acting as a bridge between both responses [53]. DCs present antigens to T cells; however, cytotoxic DCs activated by IFNα or IFNγ present antitumor activity by the TRAIL system [54]. TRAIL reduces T regulatory cells (Tregs), while increasing the CD8+ CTLs population [10].

TRAIL shares homology with FasL, another member of the TNF family that can induce T cell apoptosis. In a pro-tumoral TME, TRAIL, soluble or membrane bound, can induce apoptosis in IL2-secreting T cells but not inactivated T cells [55]. Fas ligand (FasL/CD95L) expressed by tumors allows them to inhibit T cell recognition and elimination. FasL is associated with immune escape because it binds to Fas in the T cell membrane and induces apoptotic signals. In addition, galectin-1 participates in the immunosuppressive tumor microenvironment, improving FasL activity [52]. It seems that cancer cells can release microvesicles with FAS and TRAIL, which, instead, induce apoptosis of cancer cells and target and eliminate CTLs as an immune escape mechanism. In multiple myeloma cancer cells, TRAIL bound to membranes can eliminate osteoclasts and bone formation, consequently improving the distribution of cancer cells to other tissues and allow metastasis development [10]. In metastatic tumors, cells can evade immune surveillance by inducing cell death of tumor-infiltrating lymphocytes (TILs). CRC cells expressing TRAIL can induce apoptosis of CD8+ cells by this mechanism [56]. In lymphomas, cancer cells can develop TRAIL resistance by the expression of CD40, a co-stimulatory receptor for interaction with CD4+ T cells that protects apoptosis by TRAIL. CD40 upregulates NF-kB, cFLIP, and Bcl-XL [10,31].

In this microenvironment of resistant tumor cells, TRAIL can potentiate immune suppressive effects of Tregs. Tumor-infiltrating Tregs reduce antitumor immune responses by secretion of TGFβ, IL10, and IL35, inhibiting CTL, NK cell, and DC activity. IL35 can stimulate macrophages and neutrophil polarization to an M2 anti-inflammatory state that promotes tumor development. Another cytokine that suppresses TRAIL activity in cancer cells is IL8, by upregulation of cFLIP in a CXCR2- and NF-kB-dependent way [57]. In addition, cancer cells from primary tumors release IL4, increasing the expression of anti-apoptotic proteins, such as cFLIP, Bcl-XL, and Bcl-2 [58]. It is important to note that even when cancer cells are resistant to TRAIL, exposure activates the secretion of the immune-suppressive cytokines, IL8, CXCL1, CXCL5, and CCL2 in a FADD-dependent way [59]. 

CCL2 is important because it seems to modulate the immune environment to a pro-tumoral state when interacting with cells with CCR2 receptors. As mentioned before, the interaction of cancer cells with endogenous TRAIL induces FADD-dependent secretion of CCL2, which polarizes monocytes to the M2 macrophage phenotype [59]. In addition, TRAIL receptors and FADD can promote NF-kB activation and proliferation of tumor cells. Moreover, CCL2 supports tumor growth by acting as a chemoattractant for MDSCs and monocytes, promoting its MS differentiation by their CD206 expression [28]. The reduction in TRAIL expression is stimulated by IL6, IL1β, IL17, and G-CSF through STAT3-dependent downregulation and upregulation of MMP9. The result is immune suppression and a pro-angiogenic state [10].

## 6. TRAIL Activity in Angiogenesis

Angiogenesis allows the support of tissue growth and organ function under physiological and pathological conditions. During the pathological process, such as cancer, this mechanism helps the tumor feed, supply oxygen, and eliminate waste from the body [49]. The process of generating new blood vessels occurs through several different mechanisms: (1) from pre-existing vasculature; (2) inducing new blood vessel formation through a process involving formation and outgrowth of sprouts (tip cells), which eventually fuse with an existing vessel or newly formed sprout; (3) vasculogenesis (neo-vascularization from endothelial progenitor cells); (4) vascular mimicry, in which aggressively growing tumor cells can form vessel-like structures, which are formed without the contribution of endothelial cells, and that represents an alternate channel for tumor cells to source sufficient blood supply and nutrients; and (5) trans-differentiation of CSCs (neo-vascularization in tumors through differentiation of CSCs to endothelial cells) [60]. 

Normal stem cells and CSCs grow primarily in vascular niches due to a perivascular microenvironment [61]. Tumors can be vascularized through the cooperation of endothelial cells [62]. The involvement of CSC is key to promote angiogenesis in cancer and disease progression. Studies suggest that Notch signaling plays an essential role in angiogenesis through interactions with Notch ligands to cross-talk with other pathways, such as vascular endothelial growth factor (VEGF) signaling [63]. However, the vascular niche of cancer is rich in abnormal blood vessels. These abnormalities are induced by hypoxia, low pH, and high pressure of a hostile interstitial fluid. Hypoxia also activates NF-kB and promotes EMT [64].

Furthermore, hypoxia increases hypoxia-inducible 1alpha (HIF1α) in cancer cells [65]. Moreover, hypoxia increases nitric oxide (NO), which activates the Wnt/b-catenin signaling pathway, VEGF-A, and, ultimately, angiogenesis [66]. Angiogenesis is orchestrated within the tumor mass that harbors various host-derived cells, regulated by secreted regulators, such as VEGFR2, the expression of Tie-2 monocytes, fibroblasts, endothelial cells, and innate and adaptive immune cells that are central regulators of pro-angiogenic VEGF and angiopoietin signaling [67]. The role of CD133 in angiogenesis was recently reported since it is observed that it regulates the expression of the angiogenic protein vascular endothelial growth factor (VEGF) by activating the Wnt/b-catenin signaling pathway and promoting greater recruitment of endothelial progenitor cells (CPEs) in CSC-enriched tumors. This mechanism increases VEGF-A and interleukin 8 (IL8) expression. Both factors cause neovascularization and tumor growth [60].

TRAIL can modulate angiogenesis as endothelial cells from tumor vasculature also express TRAIL receptors; this indicates that endothelial cells are sensitive to TRAIL apoptosis [68]. However, TRAIL modulates multiple cellular functions in endothelial cells involving the ECM necessary for vascular remodeling. TRAIL regulates FGF-2 angiogenic function in human endothelial cells (HMEC-1); FGF2 is a growth factor that activates endothelial cell proliferation, migration, and tubule formation [69]. Mice lacking TRAIL have increased vascular leakage. In vitro, TRAIL at low concentrations (10 ng/mL) reduces angiotensin II-induced oxidative stress, leukocyte adhesion, and permeability as it prevents redistribution of VE-cadherin from the cell membrane [70]. The effects induced by TRAIL involve NOX4, which participates in the generation of oxygen species and catalyzing the transfer of electrons from NADPH to O_2_. Via NOX4, TRAIL promotes angiogenesis by modulation of H_2_O_2_ production, eNOS phosphorylation, and NO production [71]. Low production of H_2_O_2_ from NOX4 activates MAPK family members, the TGF-β1/SMAD2/3 pathway of PI3K/AKT signaling, and cell proliferation, migration, and angiogenesis [26]. Moreover, angiogenesis induced by TRAIL can improve perfusion in ischemic disease, as TRAIL receptors are expressed by vascular smooth muscle cells and cardiomyocytes from the cardiovascular system, contributing to the pathophysiology of cardiovascular diseases. In addition, TRAIL can induce apoptosis of vascular smooth muscle cells [72]. 

Conversely, TRAIL administration has anti-angiogenic action, inducing tumor starvation and downregulation of OPG receptors [10,64]. sTRAIL confirmed its anti-angiogenetic potential, even higher compared to recombinant human TRAIL (rhTRAIL). Therefore, sTRAIL seems to have a double effect in this model generating PDAC cell death and reducing angiogenesis. Thus, TRAIL could induce apoptosis in tumoral and endothelial cells, even when TRAIL resistance develops. In cancer, anti-angiogenic therapy has been used to sensibilize cells to TRAIL. However, there exist different resistance mechanisms to anti-angiogenic agents that could inhibit TRAIL activity [73]. 

## 7. Regulation Mechanism

MicroRNAs (miRNAs or miRs) are a set of 18–24-nucleotide-long strands that can silence or downregulate the expression of their targets by base-pairing with the respective miRNA response elements found in the 3´UTR of the mRNA. In this way, there is a destabilization of the target mRNA; therefore, the efficiency of processing is reduced, which leads to an overall protein decrease [74]. What is even more remarkable is that a single miR is known to have hundreds if not thousands of targets that may be involved in many cell regulatory processes, including differentiation and apoptosis [75,76]. Not surprisingly, miRs have been shown to have different expression patterns when comparing cancerous with normal tissue, and even within cancer, malignant states often vary in expression [77]. 

TRAIL is a member of the TNF family, which, when activated, can induce apoptosis in tumor cells with no cytotoxicity to normal cells [78]. Unfortunately, many human cancer cells are resistant to TRAIL-induced apoptosis; hence, pharmacological studies have had significant drawbacks. Nonetheless, there is a silver lining as researchers are currently unraveling the different miRs involved in TRAIL regulation and TRAIL-induced apoptosis, which could, in turn, become either targets of TRAIL resistance or direct targets that induce TRAIL-induced apoptosis [78,79]. Interestingly, TRAIL resistance seems to be enhanced by PTEN and TIMP3 downregulation. To achieve this, the cluster of miR-221/222 promotes the phosphorylation of Akt, enriching the population of CD44^+^ cells, which are known to enhance invasion and tumorgenicity [27,80,81]. In addition, miR-221 can also downregulate proapoptotic, Bcl-2-modifying factor (Bmf), and p53 upregulated modulator of apoptosis (PUMA) [82]. Moreover, miR-221 has been detected in several cancer pathologies and has been identified in high levels in peripheral blood, making it an excellent biomarker for early detection [83]. We should note that although the mechanisms are not yet fully determined, it has been shown that BMF and certain energy enzymes are involved in TRAIL-induced necrosis, most likely through the TNF-R1 via activation of RIPKs, which promote mitochondrial fragmentation through MLKL and PGAM5 [69,76]. Opposing this activity, the activation of miR-125b, miR-224, and miR-122 can target Mcl-1 and Bcl-w, both anti-apoptotic factors [27,84,85].

Another interesting regulator in TRAIL apoptosis is miR-25, as it has been implicated to block TRAIL death receptor (DR) 4, thereby blocking induced apoptosis. Additionally, predictive analysis has also confirmed Bim and Mcl-1 as targets for miR-25 [86,87]. In the case of DR4, bioinformatic analysis has determined direct targeting of the 3´UTR of DR4 by miR-25 [86]. Moreover, DR4 can also be repressed by Hedgehog signaling Gl3, thereby serving as an antagonist to TRAIL-induced apoptosis [88]. miR-25 is associated with the sensitivity of liver cancer stem cells to TRAIL-induced apoptosis. Studies have reported that the knockdown of miR-25 promotes TRAIL-induced apoptosis by inhibiting the PI3K/Akt/Bad signaling pathway through the miR-25/PTEN axis. The combination of anti-miR-25 and TRAIL may represent a novel strategy for treating LCSCs [89].

PTEN has a key function in the regulation of cell survival pathways, such as the aforementioned PI3K/AKT/mTOR and MAPK pathways; its inhibition by different mi-RNAs (miR-21, miR-221, miR-23b, miR-214) has been associated with resistance to chemotherapeutic agents, as well as proapoptotic mechanisms, such as those induced by TRAIL [90]. In addition, it inhibits metastasis development, invasion, and angiogenesis [29,91,92]. Meanwhile, miR-25-3p (part of the miR-25 cluster) has been shown to promote malignant phenotypes by also regulating the PTEN/Akt pathway and the promotion of the epithelial–mesenchymal transition similarly, as does miR-92a in non-small-cell lung cancer cells (NSCLC) and miR-129-5p in retinoblastomas by targeting PAX6 [93,94,95]. In addition, Wan et al. showed that miR-25-3p can induce Vimentin and Snail and suppress E-cadherin, which enhances invasiveness [86,96]. 

MiR-148a has an interesting effect on cancer, as it has been demonstrated to both reduce tumorigenesis and induce TRAIL apoptosis. Particularly, MMP15 and ROCK1, crucial players in invasion, have been shown as direct targets of miR-148a [97]. In addition, the NF-κB/p65 pathway, which leads to TRAIL resistance, has been previously shown to be under the control of miR-30c, miR-100, and miR-21 [97,98]. Partial elucidation of the mechanism of resistance by miR-21 involves downregulation of caspase-8, which blocks receptor-interacting protein-1 cleavage; meanwhile, miR-30 involves direct binding to the 3’UTR of metastasis-associated protein-1, promoting invasion [99]. Finally, miR-100 has been shown to target mTOR, a key regulator of motility by the PI3K/Akt pathway, which leads to the regulation of 4E-BP1 and p70S6K pathways. Interestingly, p70S6K is a cell cycle effector that directly regulates mRNA in cell cycle progression [100].

TRAIL-mediated apoptosis in prostate cancer seems to correlate directly with the expression of miR-135a-3p. Shin et al. investigated the role of Tanshinone I. Their research concluded that co-treatment directly with TRAIL upregulates DR5 and miR-135a-3p. Moreover, when using miR-135a-3p mimics, PARP cleavage further increases, leading to an increase in apoptotic key regulator Bcl2-associated X protein (Bax) [101]. Still under investigation, there are several miRs of the miR-519 and miR-520 families that have been predicted to also indirectly activate proapoptotic factors Bax and Bak or enhance caspase 8 and 3 activity by FADD activity; in addition, KEGG analysis also shows that most of the targets of these families are associated with the PI3K/Akt pathway, similarly to miR-100, and although many of the hypothesized genes continue to require validation, both the NF-κB-inducing kinase and RELA have been confirmed [102,103].

## 8. Mechanism against TRAIL Resistance

TRAIL presents a limitation in its use as antitumoral therapy, as many primary tumors develop resistance to monotherapy with recombinant TRAIL and TRAIL receptor agonists [104]. Thus, the need to combine strategies to increase TRAIL sensitization and prevent resistance is clear. Table 2 shows different approaches being carried out using TRAIL as a therapeutic target in clinical trials that combine drugs or other strategies.

Targeting CSCs by TRAIL can be difficult as fast resistance development is reported. Todaro et al. presented that CD133+ CSCs from colon carcinomas can release IL4 to prevent apoptosis. However, they can be sensitized, as Loebinger et al. showed that MSC-expressing TRAIL can migrate to tumors and reduce tumor growth and metastasis of primary cancer. The combination of TRAIL plus chemotherapy with mitoxantrone increases the synergistic effect, improving apoptosis of putative CSCs. In CSCs that produce IL4, the administration of the IL4Rα antagonist of anti-IL4 neutralizing antibodies enhances the sensitivity of CD133+ cells to chemotherapy with oxaliplatin and 5-FU [105]. As another example, MSCs expressing TRAIL inhibit metastasis of the non-small-cell lung cancer (NSCLC)-derived H460 cell line combined with Claudin-7. This small molecule regulates mitogen-activated protein kinase/extracellular signal-regulated kinase (MEK/ERK) signaling pathways. Other studies have shown that targeting of the XIAP molecule increases CSC sensitivity to TRAIL in pancreatic cancer, reducing metastasis. CD133+ CSCs from brain overexpress BCL-2 after TRAIL induction, and its knockdown enhances CSC sensitivity to TRAIL. In nasopharyngeal carcinoma, the use of a second mitochondria-derived activator of caspases (SMAC) mimics the induced inhibitor of apoptosis (IAP) degradation and enhances TRAIL apoptosis. Moreover, the knockdown of Sirtuin 1 (SIR1) sensitizes CSCs from colon cancer to TRAIL cytotoxicity [106].

Some studies have shown that TRAIL-induced apoptosis is regulated by post-translational modifications of death receptors [45]. O-glycosylation of DR4 and DR5 is proven to control the sensitivity of many cancer cells to TRAIL [36]. Subsequently, Dufour et al. reported that N-glycosylated DR4 promotes TRAIL signaling [46]. We previously found that DR5 is activated by fucosylation for TRAIL-induced apoptosis using our TRAIL variants [47]. A relationship between HDAC inhibition and glycosylation patterns has been reported. This finding can be an explanation for the increased sensitivity of TRAIL receptors in the presence of HDAC inhibitors.

Epigenetic factors, such as drug resistance and immune evasion mechanisms, allow tumor progression. Histone deacetylases (HDACs) are important promoters of TRAIL resistance via TRAIL receptors. Since HDACs are associated with changes in glycosylation patterns, O-glycosylation, N-glycosylation, and fucosylation in DR4 and DR5 receptors are necessary to improve TRAIL signaling. Thus, HDAC inhibitors have been proposed as another strategy against cancer since they maintain glycosylation in TRAIL receptors [107]. In addition, HDAC inhibitors act in synergy with TRAIL by upregulating the mitochondrial pathway; downregulating NF-kβ and its gene products, such as cyclin D1, Bcl-2, Bcl-XL, VEGF, HIF-1a, IL6, IL8, MMP-2, and MMP-9; and upregulating the pro-apoptotic proteins Bax, Bak, and p21/CIP1 and TRAIL receptors DR4 and DR5 in cancer cells [108]. 

**Figure 2:**
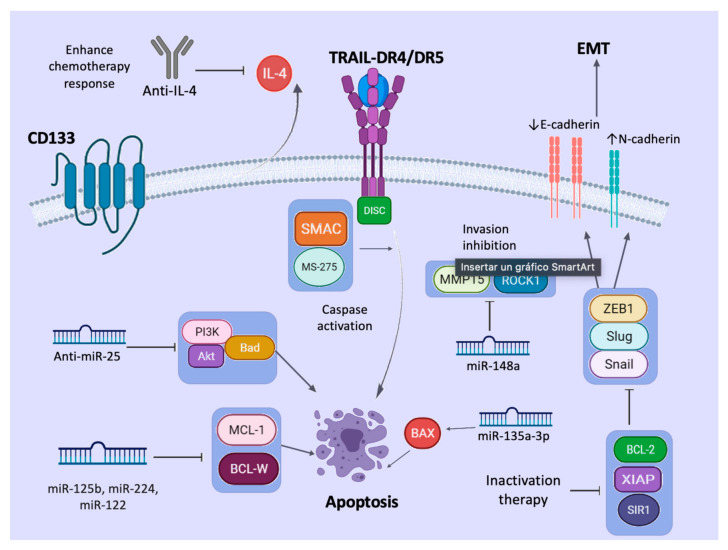
Mechanisms again TRAIL resistance. Therapeutic agents against TRAIL resistance by CSCs have been discovered. The use of anti-IL4 antibodies enhances the chemotherapy response. HDAC inhibitors, such as MS-275 and second mitochon-dria-derived activator of caspases (SMAC), sensitize CSCs resistant to TRAIL and potentiate apoptosis. Inactivation of BCL-2, SIR1, and XIAP inhibits EMT. Some miRNAs and anti-miRNAs could be used as additional strategies against cancer. Anti-miR-25 inhibits PI3K/Akt activation; miR-125b, miR-224, and miR-122 inhibit anti-apoptotic proteins MCL-1 and BCL-W; miR-135a-3p activates pro-apoptotic proteins, such as BAX; and miR-148a inhibits proteins related to inva-sion of CSCs, such as MMP15 and ROCK1. Created with BioRender.com (accessed on 25 May 2021).

It has been reported that the HDAC inhibitor MS-275 can sensitize TRAIL-resistant breast cancer xenografts in nude mice through upregulation of DR4 and DR5 TRAIL receptors, inducing apoptosis, tumor cell growth inhibition, angiogenesis, and metastasis. All these mechanisms generate a reversion of EMT, upregulate E-cadherin, and downregulate N-cadherin and transcription factors, such as Snail, Slug, and ZEB1 [108]. Moreover, the compound suberoylanilide hydroxamic acid (SAHA), another HDAC inhibitor, significantly increases the expression of Caspase-3 and the expression in MDA-MB-231 but not in MCF-7 breast cancer cells [109]. Recently, hypersensitization of CSCs to TRAIL required TRAIL-R2 and increased microenvironmental stress by the endoplasmic reticulum stress inducer celecoxib [110]. Therefore, microenvironmental modification could be a strategy to improve TRAIL sensitivity of CSCs. In addition, more research on agents that can act on CSC spheroids and thus avoid tumor progression, metastasis, and angiogenesis is needed [111].Figure 2 summarizes the different approaches that could be used against TRAIL resistance in CSCs.

## 9. Conclusions

Cancer treatments have evolved; however, cancer cells have developed several resistance mechanisms. TRAIL research demonstrates that this protein can induce tumor cell apoptosis of a wide variety of cancers when used as a recombinant TRAIL or TRAIL receptor agonist. CSC populations inside tumors have developed ways to evade this mechanism and activate survival pathways, proliferation, and angiogenesis that allow tumor progression. Likewise, CSCs can modulate the microenvironment to improve immune cell and cytokine recruitment, hypoxia, and the action of microRNAs generated by those cells. Thus, a reaction strategy has been developed that uses the combination of drugs and chemotherapeutic agents to increase CSC sensitivity to TRAIL and thereby facilitate its elimination, which reduces metastasis.

## Figures and Tables

**Table 1:** Treatments that increase TRAIL sensitivity.

Treatment	Cancer	Effect	Reference
Goniothalamin plus TRAIL	Colorectal cancer	Enhance cytotoxicity and apoptosis	[35]
Icaritin plus TRAIL	Glioblastoma	Enhance apoptosis by c-FLIP downregulation and inhibition of NF-κB activity	[36]
Micelle-in-liposomes with piperlongumine plus TRAIL	Prostate cancer	Increase sensitization to TRAIL apoptosis in cancer cells	[37]
Silibinin plus TRAIL	Glioma	Enhance apoptosis by upregulation of DR5 and downregulation of cFLIP and survival	[38]
SH122 plus TRAIL	Prostate cancer	Enhanced TRAIL-induced apoptosis via D5R and the mitochondrial pathway	[39]
MSC/dTRAIL-TK gene therapy	Renal cell carcinoma	Enhance sensitization to TRAIL and increase apoptosis	[40]
Duloxetine plus TRAIL	Lung cancer	Enhance apoptosis of tumor cells through inhibition of autophagy	[41]
3-Methyladenine and chloroquine plus TRAIL	Malignant melanoma and osteosarcoma	Enhance pro-apoptotic mitochondrial pathway of tumor cells through inhibition of autophagy	[42]
Adenovirus-p53 plus TRAIL	Ovarian and nasopharyngeal squamous cancer	Overexpression of DR5 receptor in cancer cells to increase apoptosis by TRAIL	[43]
Adenovirus E1A plus adenovirus-hTRAIL	Hepatic cancer	Enhance apoptosis by upregulation of TRAIL receptors	[44]
MiR-760 plus TRAIL	Non-small-cell lung cancer	Enhance apoptosis by targeting FOXA1	[45]

**Table 2:** Recombinant TRAIL use in clinical trials.

Recombinant TRAIL	Disease	Phase	Clinical Trial
Recombinant human Apo-2 ligand for injection	Non-small-cell lung cancer (NSCLC) stage IV	3	NCT03083743
Recombinant human TRAIL–trimer fusion protein (SCB-313)	Malignant pleural effusions	1	NCT038669697
	Peritoneal malignancies	1	NCT03443674
	Peritoneal carcinomatosis	1	NCT04047771
rhApo2L/TRAIL (AMG 951) with chemotherapy bevacizumab	Non-small-cell lung cancer (NSCLC)	2	NCT00508625
Dulanermin plus rituximab	Non-Hodgkin’s lymphoma	1, 2	NCT00400764
Dulanermin plus Camptosar^®^ /Erbitux^®^ or FOLFIRI	Metastatic colorectal cancer	1	NCT00671372
Dulanermin with FOLFOX and bevacizumab	Metastatic colorectal cancer	1	NCT00873756

## Abbreviations

ABCG5	ATP-binding cassette transporter
APAF-1	Apoptotic protease activating factor 1
AP1	Activator protein 1
Apo2L	Apo-2 ligand
Bcl-2	B-cell lymphoma 2
BPM	Bone morphogenetic protein
CAD	Caspase-activated DNase
CAF	Cancer-associated fibroblasts
CRC	Colorectal cancer
CRD	Cysteine-rich domain
CSCs	Cancer stem cells
CTLs	Cytotoxic T cells
DAMPs	Damage-associated molecular patterns
DCs	Dendritic cells
DED	Death-inducing signaling complex
DCRs	Decoy receptors
DD	Death domain
DISC	Death-inducing signaling complex
DKK-1	Dickkopf-1
DR	Death receptor
EGF	Epidermal growth factor
ECM	Extracellular matrix
EMT	Epithelial–mesenchymal transition
EMILIN2	Elastin microfibril interface-located protein 2
FADD	Fas-associated death domain
FLIP	FLICE-like inhibitory protein
FOX	Forkhead Box
HGF	Hepatocyte growth factor
HIF-1α	Hypoxia-inducible 1alpha
ICAD	Inhibitor of caspase-activated DNase
IFN-β	Interferon-β
IL8	Interleukin 8
MAPK	mitogen-activated protein kinase
MDP	membrane-proximal domain
MDSC	myeloid-derived suppressor cells
MET	mesenchymal–epithelial transition
miRNAs or miRs	microRNAs
MMP12	matrix metallopeptidase 12
MSCs	Mesenchymal stem cells
NFAT	Nuclear factor of activated T cells
NK	Natural killer
NO	Nitric oxide
NSCLC	Non-small-cell lung cancer
OPG	Osteoprotegerin
PDGF	Platelet-derived growth factor
PI3K	Phosphatidylinositol-3-kinase
PTEN	Phosphatase and tensin homolog
rhTRAIL	Recombinant human TRAIL
sTRAIL	Soluble TRAIL
TAMs	Tumor-associated macrophages
TGF-β	Transforming growth factor-beta
Tregs	Regulatory T cells
TILs	Tumor-infiltrating lymphocytes
VEGF	Vascular endothelial growth factor
TNF	Tumor necrosis factor
TRAF2	Tumor receptor-associated factor 2
TRAIL	Tumor necrosis factor (TNF)-related apoptosis-inducing ligand
TNF-α	Tumor necrosis factor-alpha
VCAM-1	Vascular cell adhesion molecule-1
VEGF	Vascular endothelial growth factor
